# Integrative Genomics Implicates EGFR as a Downstream Mediator in *NKX2-1* Amplified Non-Small Cell Lung Cancer

**DOI:** 10.1371/journal.pone.0142061

**Published:** 2015-11-10

**Authors:** Nicole Clarke, Jewison Biscocho, Kevin A. Kwei, Jean M. Davidson, Sushmita Sridhar, Xue Gong, Jonathan R. Pollack

**Affiliations:** Department of Pathology, Stanford University School of Medicine, Stanford, California, United States of America; Duke University, UNITED STATES

## Abstract

*NKX2-1*, encoding a homeobox transcription factor, is amplified in approximately 15% of non-small cell lung cancers (NSCLC), where it is thought to drive cancer cell proliferation and survival. However, its mechanism of action remains largely unknown. To identify relevant downstream transcriptional targets, here we carried out a combined NKX2-1 transcriptome (NKX2-1 knockdown followed by RNAseq) and cistrome (NKX2-1 binding sites by ChIPseq) analysis in four *NKX2-1*-amplified human NSCLC cell lines. While NKX2-1 regulated genes differed among the four cell lines assayed, cell proliferation emerged as a common theme. Moreover, in 3 of the 4 cell lines, epidermal growth factor receptor (EGFR) was among the top NKX2-1 upregulated targets, which we confirmed at the protein level by western blot. Interestingly, EGFR knockdown led to upregulation of NKX2-1, suggesting a negative feedback loop. Consistent with this finding, combined knockdown of NKX2-1 and EGFR in NCI-H1819 lung cancer cells reduced cell proliferation (as well as MAP-kinase and PI3-kinase signaling) more than knockdown of either alone. Likewise, NKX2-1 knockdown enhanced the growth-inhibitory effect of the EGFR-inhibitor erlotinib. Taken together, our findings implicate EGFR as a downstream effector of NKX2-1 in *NKX2-1* amplified NSCLC, with possible clinical implications, and provide a rich dataset for investigating additional mediators of NKX2-1 driven oncogenesis.

## Introduction

Lung cancer accounts for the largest number of cancer-related deaths in the United States [1]. There are two major classes, small cell lung cancer and non-small cell lung cancer (NSCLC), the latter representing about 85% of cases, and including adenocarcinoma, squamous cell carcinoma, and large cell carcinoma histologies [2]. Among NSCLCs, recognized cancer drivers include activating mutations in *EGFR*, *KRAS*, *BRAF* and *ERBB2* (HER2), as well as rearrangements of *ALK* and *ROS1* [3]. Some of these, only recently identified, are now valuable therapeutic targets, underscoring the importance of defining new molecular targets and mechanisms.

*NKX2-1* (also called *TITF1* and *TTF-1*) encodes a homeobox transcription factor, and is found amplified at cytoband 14q13 in about 15% of NSCLCs (predominantly adenocarcinomas) [4, 5]. Understanding its mechanisms may provide new insight into lung carcinogenesis, and new strategies for therapy. NKX2-1 is expressed in the normal developing lung (as well as thyroid and forebrain) [6], where it is essential for organogenesis. In the adult lung, NKX2-1 expression is restricted to club cells (Clara) and type II pneumocytes, where it regulates surfactant production.

For years, NKX2-1 expression has been used by pathologists to deduce a lung origin of carcinomas [7]. Recently, *NKX2-1* has been linked more directly to lung cancer, where the gene locus is amplified in some cases, leading to enhanced lung cancer cell proliferation and survival [8–11]. While its dual roles in driving lung development and differentiation on the one hand, and lung cancer (often viewed as de-differentiation) on the other seem paradoxical, NKX2-1 fits well into an emerging class of “lineage-survival” oncogenes—often master transcriptional regulators of normal cell lineage that become deregulated in cancers derived from that lineage [12]. Other examples include androgen receptor (AR) in prostate cancer, and MITF in melanoma.

Recent studies have identified candidate downstream mediators and collaborators of NKX2-1 oncogenesis, including ROR1 [13] and LMO3 [14]. Nonetheless, the mechanisms by which NKX2-1 contributes to lung carcinogenesis remain largely unknown. Indeed, in some contexts (mainly in the mouse), Nkx2-1 seems to function more as a tumor suppressor, inhibiting Kras-driven lung cancer and lung cancer metastasis [15, 16]. Here, to uncover oncogenic mechanisms in human lung cancer, we carried out a combined transcriptome (NKX2-1 knockdown followed by RNAseq) and cistrome (NKX2-1 binding sites by ChIP-seq) analysis in *NKX2-1* amplified NSCLC cell lines. Among our findings, we identify EGFR as a downstream effector of NKX2-1 driven cell proliferation. Our results provide new insight into the mechanisms of NKX2-1 mediated lung cancer, and a dataset for continued exploration.

## Materials and Methods

### Cell culture

NCI-H1819, NCI-H661, HCC1195 and HCC1833 cell lines were obtained from Dr. John Minna (University of Texas Southwestern Medical Center) [17–20], where they were authenticated by short tandem repeat analysis. All cell lines were grown at 37°C in RPMI-1640 medium with L-glutamate, supplemented with 10% (vol/vol) fetal bovine serum and 1X Pen/Strep.

### NKX2-1 isoform expression

A full-length NKX2-1 cDNA clone (in pOTB7) was obtained from Origene. DNA constructs corresponding to NKX2-1 transcript variant 1 (NM_001079668.2; encoding 401 amino acids) and NKX2-1 transcript variant 2 (NM_003317; encoding 371 amino acids, absent the N-terminal 30 amino acids of isoform 1) were PCR-amplified from the Origene plasmid template, sequence-verified, and then subcloned into the BamHI and XhoI sites of pCDNA6 (Invitrogen). PCR primers were: variant 1-F TCGAGGATCCCATGTGGTCCGGAGGCAG; variant 2-F TCGAGGATCCCATGTCGATGAGTCCAAAGCAC; variant 1/2-R GATCCTCGAGTCACCAGGTCCGACCG. Expression constructs were transfected into 293T cells (American Type Culture Collection) using Lipofectamine 2000 (Life Technologies) following the manufacturer’s protocol, and cell lysates collected 48 hrs later.

### siRNA transfection

For siRNA transfection, 25,000–100,000 cells per 6-well plate well or 750,000–1,500,000 cells per 10cm plate were seeded and transfected using Lipofectamine 2000 (Life Technologies) following the manufacturer’s protocol. All siRNAs were On-TARGETplus pools purchased from Dharmacon/GE Healthcare (Table A in S1 File) and transfected at a final siRNA concentration of 50nM (unless otherwise specified) for 16 hr.

### Western blot

Cells were lysed in RIPA buffer (Millipore) supplemented with 1mM sodium orthovanadate, 5mM NaF, 1mM PMSF, and 1X protease inhibitor cocktail (Roche). Then 40ug lysate was run on a 4–12% polyacrylamide gel (Biorad) and transferred to PVDF membrane (Biorad). Primary antibodies used were NKX2-1 (Santa Cruz Biotechnology, H-190, 1:200), EGFR (Cell Signaling, D38B1, 1:1000), MAPK (Erk1/2) (Cell Signaling, 137F5, 1:1000), p-MAPK (Erk1/2) (Thr202/Tyr204) (Cell Signaling, D12.14.4E, 1:1000), AKT (Cell Signaling, C67E7, 1:1000), p-AKT (Ser473) (Cell Signaling, D9E, 1:1000), and α-tubulin (Santa Cruz Biotechnology, 1:1000). All secondary antibodies were HRP-conjugated and SuperSignal West Pico Chemiluminescence (Pierce) used for detection. Western blots were quantified using ImageJ. All western blot results are representative of multiple independent experiments.

### Cell proliferation/viability assay

Cell proliferation/viability was quantified 0, 24, 48, 72 and 96 hrs post-transfection by colorimetric assay based on the metabolic cleavage of the tetrazolium salt WST-1 in viable cells, according to the manufacturer protocol (Roche). In some experiments, erlotinib (Santa Cruz Biotechnology) was added at indicated concentrations (or vehicle control). IC_50_ values were determined by fitting a nonlinear log (inhibitor) versus response curve using GraphPad Prism.

### RNAseq

Cell lines were transfected with either a NKX2-1-targeting siRNA pool or a non-targeting control (NTC) siRNA pool for 16 hrs. Total protein (to verify NKX2-1 knockdown) and RNA (by Qiagen RNAeasy Mini) were collected 40 hrs post-transfection. qRT-PCR was done first to verify reduced transcript for NKX2-1, as well as a known NKX2-1 regulated gene, SFTPB [21]. For q-RT-PCR, cDNA was made using SuperScript II (Life Technologies), and then qPCR was done in at least triplicate using TaqMan reagents (Applied Biosystems) on an ABS Fast 7500 instrument. Relative transcript levels were calculated by the ΔCT method, and normalized to GAPDH. Primers used are listed in Table B in S1 File. Barcoded cDNA libraries were then prepared from total RNA using the Illumina TruSeq RNAseq kit, and sequenced (single-end 36-bp reads) on an Illumina HiSeq instrument to a depth of 24–63 million reads per sample (Stanford Center for Genomics and Personalized Medicine) (Table C in S1 File). Sequence reads were mapped to the RefSeq transcriptome and transcripts quantified as reads per kilobase per million reads (RPKM) using DNAnexus. Subsequent analysis was confined to adequately expressed transcripts, defined as RPKM ≥1 in both the NKX2-1 and control knockdowns. Gene Set Enrichment Analysis was done using the desktop package [22]. The complete dataset of raw RNAseq reads is available at the NCBI Sequence Read Archive (Accession SRP045118).

### Q-RT-PCR

For select genes, RNAseq results were subsequently validated by Q-RT-PCR. Reverse transcription was performed using SuperScript II reagents as per the manufacturer’s protocol (Life Technologies). qPCR was done in at least triplicate using TaqMan or SYBR Green reagents (Applied Biosystems) on an ABS Fast 7600 instrument. Relative transcript levels were calculated by the ΔCT method, and normalized to GAPDH. Primers used are listed in Table B in S1 File.

### Chromatin immunoprecipitation (ChIP) seq

Five million cells per immunoprecipitation (IP) or control input sample were cross-linked in 1% formaldehyde at 25°C for 10 min, washed in ice cold PBS, lysed in cell lysis buffer (5mM PIPES (pH8), 85mM KCl, 1% Igepal, 1X Roche Complete Mini Protease Inhibitor Cocktail) at 4°C for 15 min, mechanically homogenized with 25 strokes of a dounce homogenizer, lysed in nuclear lysis buffer (50mM Tris (pH8), 10mM EDTA (pH8), 1% (w/v) SDS, 1X Roche Complete Mini Protease Inhibitor Cocktail) 30 min and sonicated with a Bioruptor XL to obtain 200-600bp chromatin fragments. Sonicated fragments were diluted 10-fold in IP buffer (20mM Tris (pH8), 2mM EDTA (pH8), 2% Triton X-100, 0.2% Sodium Deoxycholate, 200mM NaCl) and incubated overnight with a 1:1 mix of Protein A/Protein G beads blocked (5mg/mL BSA, 1X Roche Complete Mini Protease Inhibitor Cocktail) and pre-incubated with 5μg anti-NKX2-1 antibody (Santa Cruz Biotechnology, H-190). For ChIP-PCR experiments, rabbit IgG (Santa Cruz Biotechnology, sc-2027) was used as a negative control. Samples were washed 3x in IP wash buffer (50mM Tris (pH8), 150mM NaCl, 0.05% Triton X-100), once in TE buffer, and eluted from the beads into IP Elution buffer (1% SDS, 100mM NaHCO_3_) at 65°C for 1 hr. Crosslinks were reversed by incubation at 65°C for 16 hrs in modified elution buffer (1% SDS, 100mM NaHCO_3_, 200mM NaCl). ChIP’ed DNA was purified using a Qiagen PCR clean up kit. To first validate ChIP antibody specificity, qPCR was performed with custom TaqMan probes designed to a known NKX2-1 binding site (*SFTPB* promoter) [21], in comparison to a negative control (an irrelevant gene, *WNT5A*), and normalized to GAPDH. Primers used are listed in Table B in S1 File. Barcoded ChIP and input DNA libraries were then prepared using the Illumina TruSeq ChIPseq kit, and then sequenced (single-end 36-bp reads) on an Illumina HiSeq instrument to a depth of 18–60 million reads per sample (Stanford Center for Genomics and Personalized Medicine) (Table C in S1 File). Sequence reads were mapped to the human genome (NCBI36, hg18) and significant binding peaks called using DNAnexus. Binding peaks were annotated to the nearest gene (within 100kb) using a custom Perl script. MEME [23] was used for *de novo* motif scanning of NKX2-1 binding peaks (100bp centered on the top 500 binding peaks associated with genes). Enrichment of canonical pathways was evaluated by the Molecular Signatures Database [24] “compute overlap” function. The complete dataset of raw ChIPseq reads is available at the NCBI Sequence Read Archive (Accession SRP045118).

### Integration of TCGA data

Processed TCGA lung adenocarcinoma [25] (n = 488) data, including DNA copy number (Affymetrix SNP 6.0) and transcript levels (RNAseq), were accessed from the Broad Firehose pipeline. Cases with *NKX2-1* amplification were defined by *NKX2-1* tumor/normal ratios > 1.5, and absence of amplification by tumor/normal <1.1. NKX2-1 high-expression was defined as the top 25 percentile of specimens. Genes differentially expressed between *NKX2-1* amplified/highly-expressed (n = 30) and non-amplified/highly-expressed (n = 60) were identified by two-class SAM analysis [26], using a false discovery rate (FDR) <0.05.

## Results

### RNAseq analysis of the NKX2-1 regulated transcriptome

**Fig 1:**
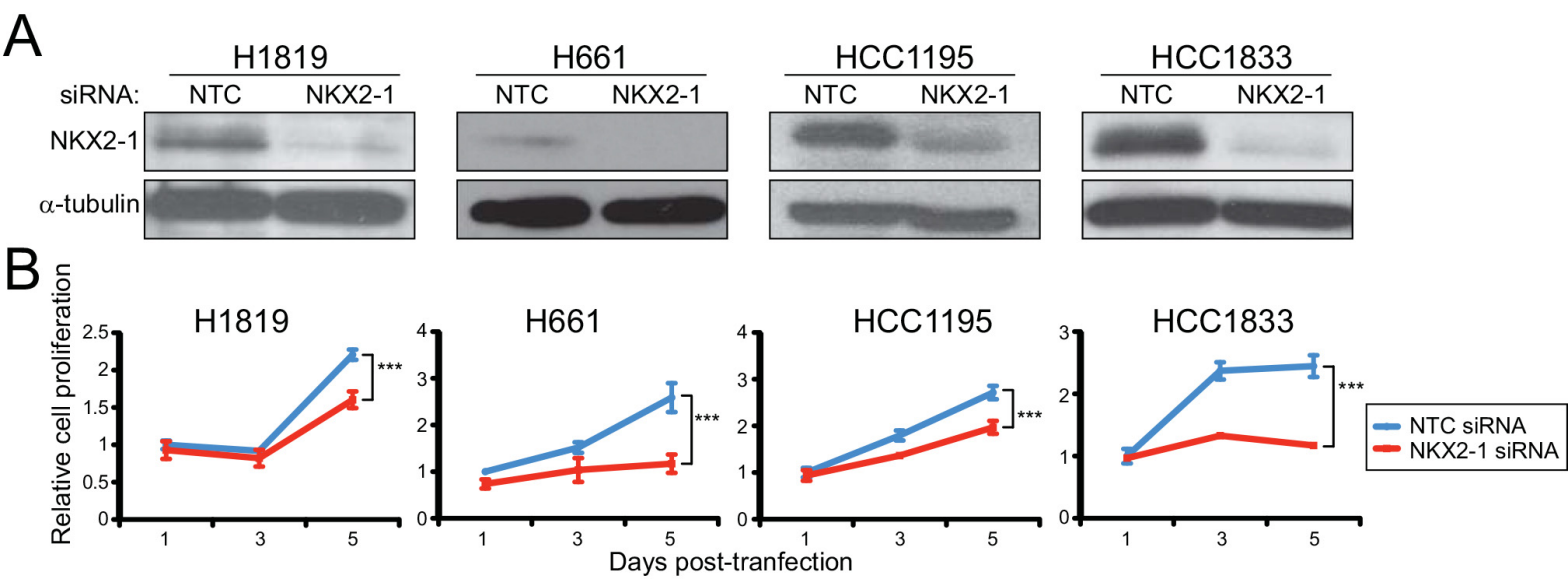
Characterization of *NKX2-1* amplified and growth-dependent NSCLC cell lines. (A) Efficient siRNA-mediated NKX2-1 knockdown in four NSCLC cell lines, demonstrated by western blot. NTC, non-targeting control. α-tubulin serves as a loading control. (B) NKX2-1 knockdown leads to significantly reduced cell proliferation, measured by WST-1 viability assay. ***, *P*-value < 0.001 (two-tailed Student’s t-test).

To investigate mechanisms of NKX2-1 mediated oncogenesis, we first sought to identify NKX2-1 regulated genes in *NKX2-1* amplified NSCLC cell lines. We previously characterized two NSCLC cell lines, HCC1195 and HCC1833, that harbor *NKX2-1* amplification and are dependent on NKX2-1 (based on knockdown studies) for cell proliferation [10]. Here, we surveyed additional NSCLC lines [19, 27], and identified two more *NKX2-1* amplified and dependent lines, H1819 and H661, for a total of four cell lines (Fig 1). Three of the four cell lines derive from lung adenocarcinomas (H1819, HCC1195, HCC1833) while the fourth (H661) is of large cell carcinoma origin. Analysis of two lines (HCC1195 and HCC1833) indicated that the shorter of two reported NKX2-1 isoforms (with variant N-termini) [6] was the one predominantly expressed (Figure A in S2 File).

**Fig 2:**
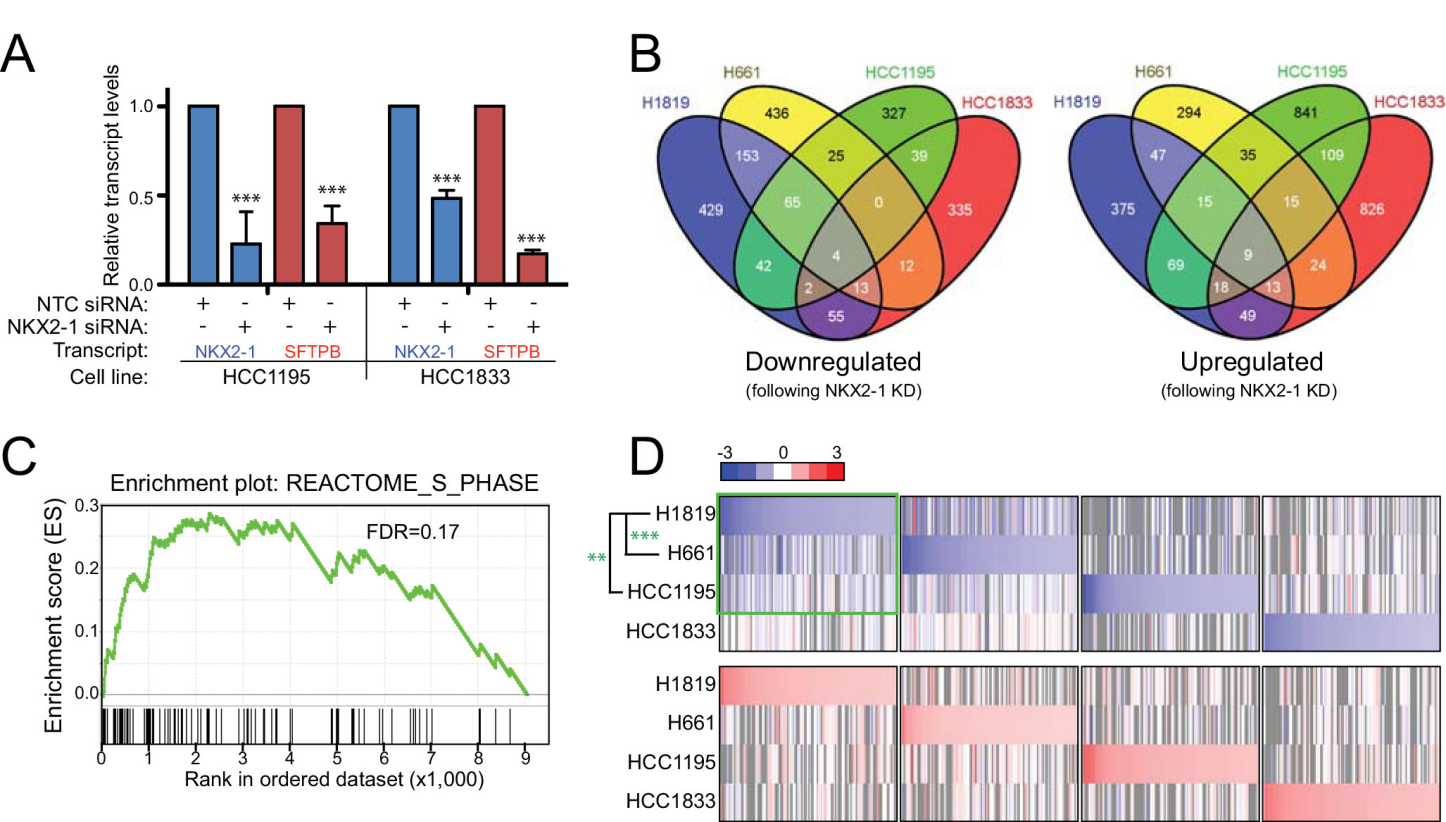
Surveying the NKX2-1 regulated transcriptome by RNAseq. (A) Validating the approach: NKX2-1 knockdown leads to reduced expression (by Q-RT-PCR) of the known NKX2-1 regulated target, SFTPB. ***, *P*-value < 0.001 (two tailed Student’s t-test). (B) Overlap among the four NSCLC cell lines of genes substantially (≥25%) down or upregulated following NKX2-1 knockdown. (C) Across the four NSCLC cell lines, GSEA reveals significant enrichment of proliferation gene sets; representative GSEA plot shown (see also Table G in S1 File). (D) Heatmap of top downregulated (blue) and upregulated (red) genes, following NKX2-1 knockdown in the four NSCLC cell lines. Note the shared downregulated expression patterns in H1819, H661 and HCC1195 (highlighted by green box). ***, *P*-value < 0.01; ***, *P*-value < 0.001 (Pearson correlation *P*-value, corrected for multiple test comparisons).

To identify NKX2-1 regulated genes in each of the four cell lines, we transfected the cells with an On-TARGETplus siRNA pool to knock down NKX2-1, or a non-targeting control (NTC) siRNA pool. (Because we had previously shown that independent siRNAs targeting NKX2-1 exhibited NKX2-1 knockdown and reduced cell proliferation comparable to the NKX2-1 siRNA pool [10] (Figure B, Panels A, B and C in S2 File), most experiments were done using the siRNA pool, with subsequent validation of select key findings using independent siRNAs.) We then assayed resultant transcriptome changes by RNAseq, comparing control and NKX2-1 knockdown cells. Transcriptomes were assayed 40 hrs post siRNA-transfection, a time point at which we observed knockdown of both the NKX2-1 transcript and protein, as well as reduced transcript of surfactant protein B (SFTPB), a known NKX2-1 regulated target [21] (Fig 2A).

Confining the analysis to well-measured genes (RPKM ≥1), approximately 10,000 genes were expressed in each of the cell lines, with 9,041 genes common across all four cell lines (Figure C, Panel A in S2 File, and Table D in S1 File). The number of genes substantially altered by NKX2-1 knockdown, defined as at least 25% decreased or increased expression, ranged from 1,160 to 1,615 among the four cell lines (Figure C, Panel B in S2 File, and Table E in S1 File). Despite this, few genes by that criterion were consistently altered across all four cell lines. Upon NKX2-1 knockdown, only 4 genes were consistently downregulated (*NBL1*, *UNC84B*, *RRBP1*, and *FA2H*), and only 9 genes were consistently upregulated (*C7ORF60*, *CHD7*, *HIST1H2BC*, *HIST1H2BD*, *HIST1H2BE*, *ID3*, *PIK3CB*, *TP53INP1*, *ZBTB4*) (Fig 2B). These genes have diverse cellular roles (Table F in S1 File), among which RRBP1 functions in endoplasmic reticulum stress response and has been found upregulated in lung cancer [28], and overexpression of the ID3 transcriptional repressor has been reported to reduce lung cancer growth *in vivo* [29].

To explore themes rather than individual genes, we also carried out a two-class gene set enrichment analysis (GSEA) [24], comparing transcript levels (across all four cell lines) following NKX2-1 knockdown *vs*. non-targeting control. The top enriched conical pathways included several relating to cell-cycle and cell proliferation (Fig 2C, and Table G in S1 File), consistent with the known role of NKX2-1 in cell proliferation [10, 30]. GSEA assessment of each cell line individually also showed enrichment of biology relating to cell proliferation (Table H in S1 File).

Although few genes met the stringent threshold for altered expression across all four cell lines (only 4 consistently downregulated and 9 upregulated genes), we investigated whether more genes might show a similar trend. Examining the top 100 downregulated and upregulated genes in each cell line (Table I in S1 File), a heatmap visualization (Fig 2D) revealed shared expression patterns among 3 of the 4 cell lines (H1819, HCC1195 and H661), particularly among genes downregulated with NKX2-1 knockdown. This finding, substantiated by correlation analysis (Fig 2D), suggested that these three cell lines might best model *NKX2-1* amplified NSCLC.

Focusing on the three cell lines (H1819, HCC1195 and H661) with shared expression patterns, 69 genes were consistently downregulated (at least 25%) upon NKX2-1 knockdown (Fig 2B, and Table J in S1 File), indicating that NKX2-1 positively regulates their expression. Although several were of possible biologic interest, notable among them was epidermal growth factor receptor (EGFR), ranked respectively 8^th^ (of 763), 68^th^ (of 504), and 18^th^ (of 708) among genes downregulated upon NKX2-1 knockdown in the three cell lines. Reduced expression of EGFR, as well as other select genes (to substantiate RNAseq results), was verified by Q-RT-PCR (Figure C, Panel C in S2 File). Two independent siRNAs targeting NKX2-1 each also reduced EGFR transcript levels (by Q-RT-PCR; Figure B, Panel D in S2 File) and protein levels (by western blot; Figure B, Panel E in S2 File), supporting EGFR reduction to be an on-target RNA interference phenotype; other candidate NKX2-1 regulated genes were not similarly evaluated. We had not previously succeeded in rescue of NKX2-1 knockdown by ectopic expression of NKX2-1 (unpublished), possibly due to non-optimal NKX2-1 expression levels. Therefore, we did not attempt to further verify on-target RNA interference by rescuing the NKX2-1 knockdown effect on EGFR levels.

### ChIPseq analysis of the NKX2-1 cistrome

**Fig 3:**
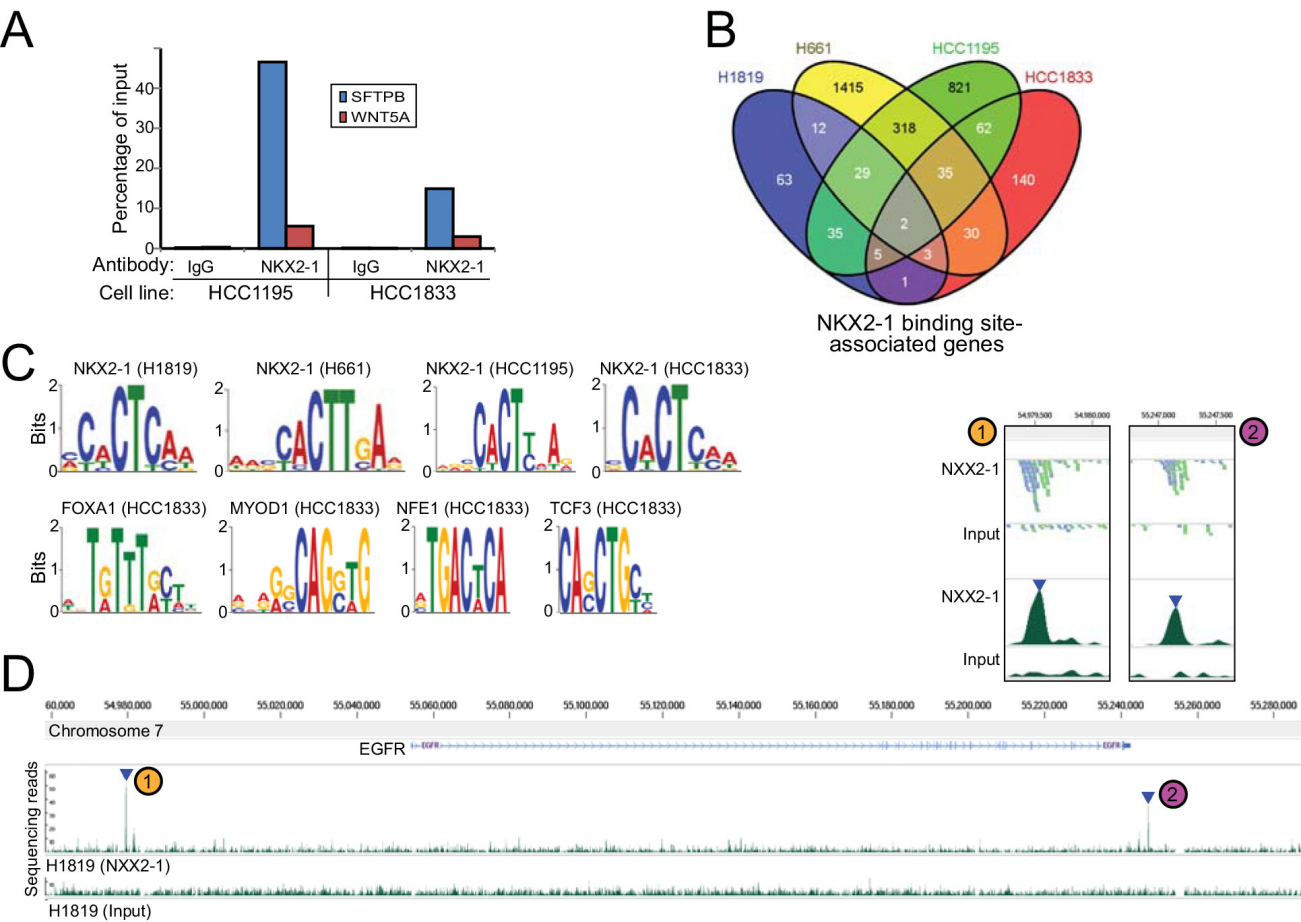
Defining the NKX2-1 cistrome by ChIPseq. (A) Validating the NKX2-1 antibody for ChIP: ChIP-PCR identifies the known NKX2-1 binding site in the SFTPB promoter. Note the enrichment of SFTPB compared to an irrelevant gene (WNT5A). (B) Overlap among the four NSCLC cell lines of NKX2-1 binding site-associated genes (within 100Kb). (C) *De novo* motif analysis re-discovers the known NKX2-1 consensus binding motif, and identifies enrichment of other transcription factor binding motifs nearby NKX2-1 binding sites. (D) NKX2-1 binding peaks identified at the EGFR locus in H1819 cells. The two called peaks are identified by blue triangles, and supporting reads are shown in the close-up inset. Binding peaks at EGFR in other cell lines are shown in Figure D, Panel B in S2 File.

While the above strategy (NKX2-1 knockdown coupled with RNAseq) revealed NKX2-1 regulated genes, it did not distinguish direct from indirect NKX2-1 transcriptional targets. Therefore, in parallel, we carried out chromatin immunoprecipitation (ChIP)-seq to define NKX2-1 genome binding sites and associated genes in the same four *NKX2-1* amplified/dependent NSCLC cell lines. ChIP-PCR of the *SFTB* promoter was done prior to ChIP-seq to validate the specificity of the anti-NKX2-1 antibody for ChIP (Fig 3A).

In the four cell lines, ChIPseq identified 150–1,844 NKX2-1 binding site-associated genes (nearest gene within 100Kb) (Fig 3B, and Table K in S1 File). Most binding sites occurred within 100Kb of genes, as would be expected for promoters or enhancers (Figure D, Panel A in S2 File). *De novo* motif analysis recovered the known Nkx2 binding site (CACTY) [31] (Fig 3C), validating the ChIPseq data quality. In addition, motif analysis of NKX2-1 binding peaks identified nearby enrichment of the FOXA1 motif in HCC1833 cells, consistent with the reported interaction of NKX2-1 with FOXA1 [32]. Other NKX2-1 binding site associated transcription factor motifs in HCC1833 included MYOD1, NFE2 and TCF3 (Fig 3C).

Despite the large number of NKX2-1 binding sites, only a small subset (2 associated genes: PVT1 and ZC3H7A) was identified across all four *NKX2-1* amplified cell lines (Fig 3B), mirroring the findings of the transcriptome analysis. Of note, NKX2-1 binding peaks were found associated with *EGFR* in all three cell lines (H1819, H661 and HCC1195) sharing similar transcriptional responses to NKX2-1 knockdown (Table K in S1 file, Fig 3D, and Figure D, Panel B in S2 File). NKX2-1 binding site motifs could be identified within the ChIPseq binding peaks at *EGFR* (Figure D, Panel B in S2 File). When considered across all cell lines together, genes nearest (and within 100Kb) to NKX2-1 binding sites were enriched for specific canonical pathways, including MAPK signaling, axon guidance, focal adhesion, and cell-cell communication (Table L in S1 File), possibly reflecting NKX2-1’s dual roles in cell proliferation and epithelial differentiation.

### Integrative -omic analysis identifies EGFR as downstream effector of amplified *NKX2-1*

To identify direct transcriptional targets of NKX2-1, we integrated the above RNAseq and ChIPseq datasets. Among the four different cell lines, 2–13% of genes whose expression changed ≥25% upon NKX2-1 knockdown also had nearby (within 100Kb) NKX2-1 binding peaks (Tables M and N in S1 File, and Figure E in S2 File). Conversely, 6–17% of genes associated with NKX2-1 binding sites exhibited substantial expression changes (≥25%) following NKX2-1 knockdown. To further whittle down the list of interesting candidates, and to ensure relevance to primary NSCLC samples, we cross-compared our list of genes to transcriptome data of 488 primary lung adenocarcinomas from The Cancer Genome Atlas (TCGA) [25]. In particular, we identified the subset of NKX2-1 upregulated presumptive direct targets whose expression was significantly higher (by two-class SAM analysis) in TCGA lung adenocarcinomas with *NKX2-1* amplification/overexpression (n = 20), compared to adenocarcinomas without amplification (but still expressing NKX2-1 in a non-amplified context) (n = 60) (Table 1). Notable among the remaining candidates was EGFR, which in summary was downregulated with NKX2-1 knockdown in 3 of 4 cell lines, was associated with a called ChIP peak in the same three cell lines, and was significantly overexpressed (FDR = 0.054) in primary NSCLCs with *NKX2-1* amplification/overexpression. Decidedly, our subsequent follow-up experiments focused on EGFR.

**Table 1:** Integration of RNAseq, ChIPseq and TCGA data.

Cell line	Gene	RNAseq (siNKX2-1/siNTC)	ChIPseq peak rank	TCGA FDR^a^ (%)
**H1819**	EGFR	0.349	84	5.4
	EFR3B	0.716	123	1.1
**H661**	EGFR	0.397	1638	5.4
	PAX9	0.632	1400	0.3
	MRPL42	0.644	1922	0.3
	TSEN15	0.644	2163	3.0
	RAI14	0.649	1989	9.2
	MAP2K6	0.667	2523	0.3
	LOC654342	0.696	2906	3.6
	GNG4	0.696	1070	5.4
	MED30	0.714	1551	0.8
	RALA	0.722	3122	1.1
	MCTP1	0.733	261	7.8
	ARL4A	0.750	401	9.2
**HCC1195**	CCL20	0.465	908	7.8
	CNGA3	0.478	1542	7.8
	EGFR	0.568	1388	5.4
	TREM1	0.571	1350	6.4
	MMP13	0.573	1367	9.2
	ETV5	0.600	437	6.4
	PENK	0.677	1564	9.2
	IL1RAP	0.683	1330	5.4
	NKX2-8	0.691	978	2.6
	ABCA4	0.691	328	5.4
	NAMPT	0.709	1176	1.5
	HMGA2	0.712	1148	4.4
	ASPHD2	0.714	1199	1.5
	C5orf4	0.739	347	9.2
	EFR3B	0.744	537	1.1
	LPIN1	0.745	698	5.4
	FAM101A	0.750	275	1.1
**HCC1833**	NGF	0.513	7	4.4
	CCDC14	0.622	202	0.3
	HMP19	0.654	216	7.8
	HMGA2	0.676	193	4.4
	BCL2	0.706	95	0.6
	ESPL1	0.741	184	0.0

^a^Amplified/highly-expressed *vs*. non-amplified/highly-expressed (FDR<10%)

**Fig 4:**
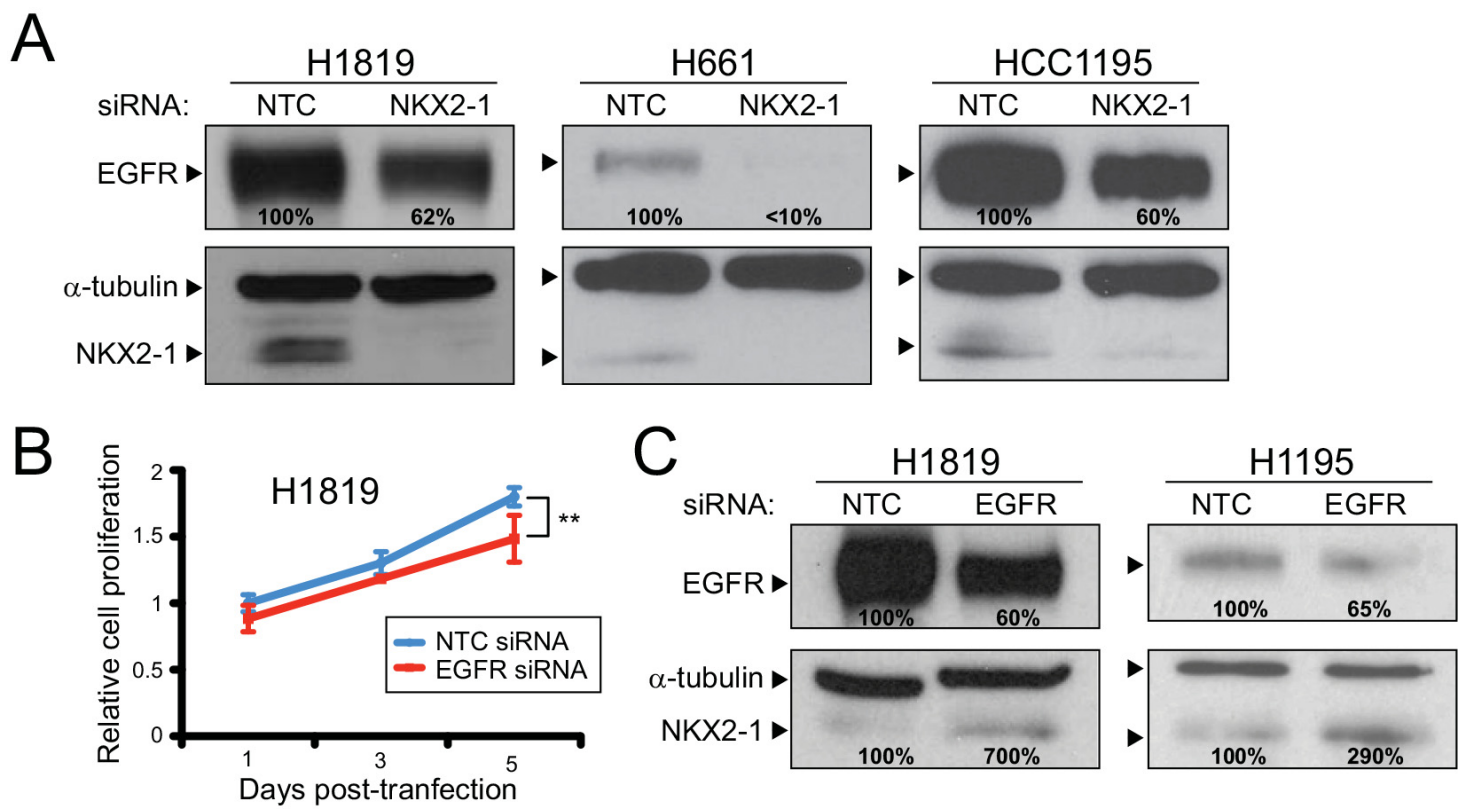
NKX2-1 regulates EGFR levels, with negative feedback. (A) NKX2-1 knockdown leads to reduced EGFR protein levels quantified by western blot (% residual indicated). Levels normalized to α-tubulin loading control. (B) EGFR knockdown by siRNA reduces cell proliferation comparable to NKX2-1 knockdown (see Fig 1A). **, *P*-value < 0.01 (two tailed Student’s t-test). (C) EGFR knockdown leads to elevated NKX2-1 protein levels (% increase indicated; levels normalized to α-tubulin loading control), suggesting negative feedback regulation.

*EGFR* is moderately amplified in H1819 cells, and is wildtype (i.e. no activating mutations) in all four lines [33–36]. EGFR protein expression varies, but was highest in H1819 (Figure F, Panel A in S2 File). Consistent with the RNAseq results, in all three cell lines (H1819, HCC1195 and H661) where NKX2-1 knockdown led to reduced EGFR transcript, NKX2-1 knockdown also led to a 40–90% reduction of EGFR protein, assessed by western blot (Fig 4A). Consistent with EGFR possibly functioning as a downstream effector of NKX2-1, knock down of EGFR (using 200nM siRNA [37]) led to a comparable reduction in cell proliferation, measured by WST-1 assay (Fig 4B). Unexpectedly, knockdown of EGFR led to increased protein levels of NKX2-1 (Fig 4C), suggesting negative feedback regulation. Two independent siRNAs targeting EGFR (Figure B, Panels F and G in S2 File) each also increased NKX2-1 protein levels (by western blot; Figure B, Panel H in S2 File), supporting an on-target RNA interference phenotype. Interestingly, EGFR knockdown did not alter NKX2-1 transcript levels (Figure B, Panel I in S2 File), suggesting that the increased NKX2-1 protein levels observed were likely the consequence of post-transcriptional regulation.

**Fig 5:**
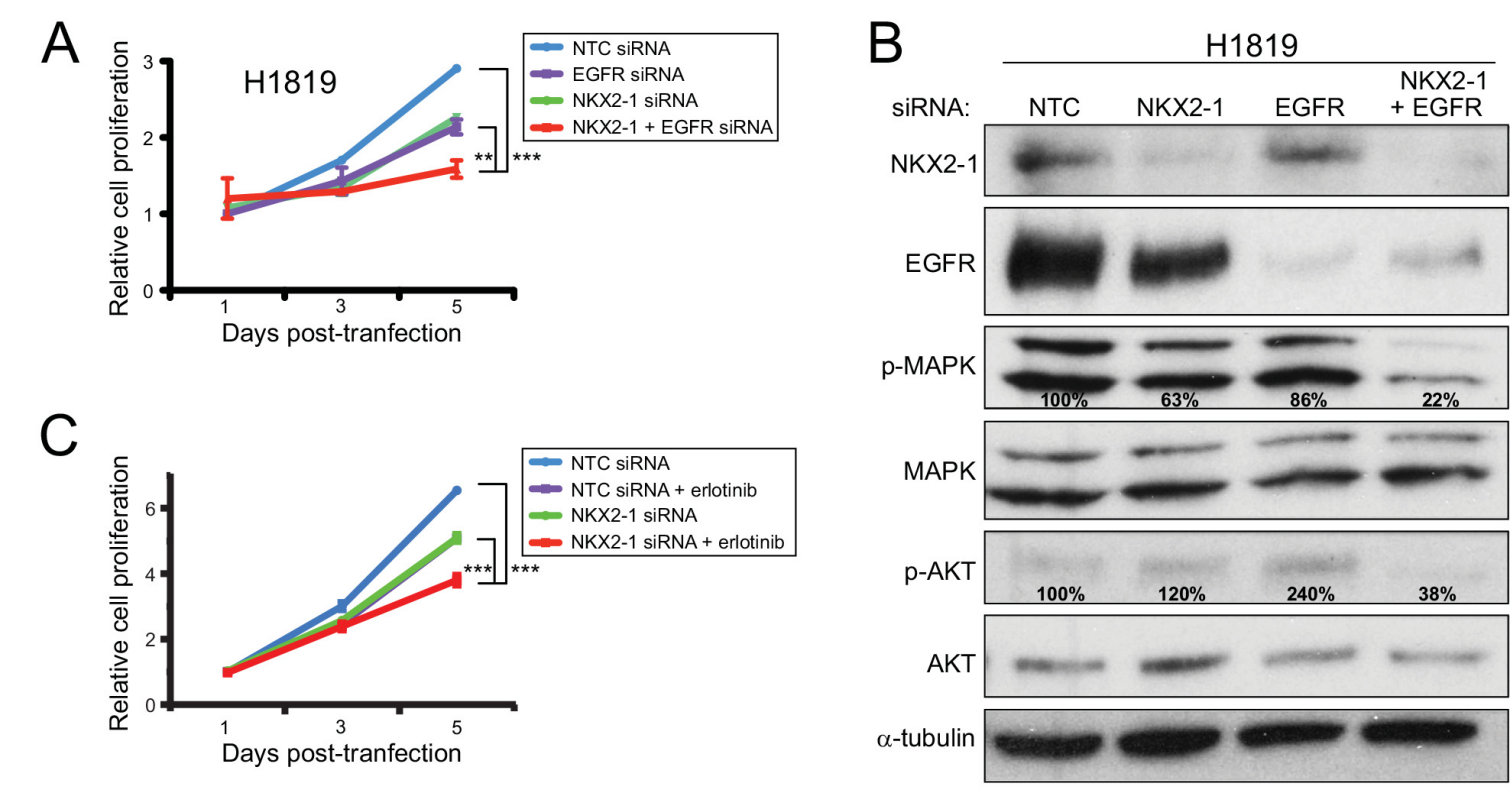
Combined NKX2-1 and EGFR knockdown reduces cell proliferation and MAPK/PI3K signaling. (A) Combined knockdown of NKX2-1 and EGFR reduces H1819 cell proliferation more than either alone. **, *P*-value < 0.01; ***, *P*-value < 0.001 (two tailed Student’s t-test). (B) Combined knockdown of NKX2-1 and EGFR in H1819 cells diminishes MAPK signaling (p-MAPK) and PI3K signaling (p-AKT) more than either alone. Percent residual indicated; levels normalized to α-tubulin loading control. Note, in the particular western shown, EGFR knockdown does not appear to increase NKX2-1 levels appreciably, although the increase has been reproducibly observed in multiple other experiments (e.g. Fig 4C, and Figure B, Panel H in S2 File). (C) NKX2-1 knockdown collaborates with EGFR inhibitor erlotinib to inhibit H1819 cell growth ***, *P*-value ≤ 0.001 (two tailed Student’s t-test).

Consistent with a negative feedback loop, knock down of NKX2-1 together with EGFR reduced H1819 cell proliferation significantly more than knockdown of either alone (Fig 5A). (The combined knockdown could not be tested in HCC1195 and H661 cells, because the higher siRNA concentration required to knockdown EGFR was toxic to those cells.) Furthermore, combined NKX2-1 and EGFR knockdown reduced MAP-kinase (p-MAPK levels) and PI3-kinase signaling (p-AKT levels)—known growth/survival signaling pathways downstream of EGFR [38]—more than knockdown of either alone (Fig 5B).

The above results suggested that NKX2-1 knockdown might collaborate with small molecule inhibition of EGFR kinase. To test this, we challenged H1819 cells with NKX2-1 knockdown (or non-targeting control) to a ~50% growth inhibitory concentration (1.0 μM; Figure F, Panel B in S2 File) of the EGFR inhibitor erlotinib. Notably, NKX2-1 knockdown significantly enhanced the growth inhibitory effect of erlotinib (Fig 5C) on the *NKX2-1* amplified NSCLC cells.

## Discussion

NKX2-1 is a transcription factor, so its oncogenic effects when amplified are presumed to be mediated through transcriptional regulation of key downstream targets. To identify those targets, here we performed a combined transcriptome (NKX2-1 knockdown followed by RNAseq) and cistrome (NKX2-1 bound genes by ChIPseq) analysis in four NSCLC cell lines exhibiting *NKX2-1* amplification and growth-dependency. Results were further integrated with TCGA genome and transcriptome data from primary NSCLC cases, from which we identified and further studied EGFR as a downstream target.

An unexpected finding from the knockdown/RNAseq experiments was the relatively small overlap of substantially up/downregulated genes across the cell lines. This likely reflects the heterogeneity among different patient’s tumors, where distinct cell types of origin, differentiation patterns, and/or tumor (epi)genetic alterations may influence the landscape of genes regulated by NKX2-1. Despite this, 3 of the 4 cell lines shared meaningful gene expression patterns, exhibited enrichment for select processes (e.g. cell proliferation), and nominated specific genes (EGFR).

The combined analysis of NKX2-1 binding sites further identified the subset of likely direct NKX2-1 transcriptional targets (though we note that indirect transcriptional targets may also have important biological functions). Finally, the integration with TCGA data highlighted those direct targets with likely relevance to actual primary tumors. While EGFR emerged as the most interesting candidate, other genes with intriguing connections to lung cancer biology, including HMGA2, BCL2, and others (Table 1) [15, 39], will be the focus of future studies. Also notable was the absence of ROR1 and LMO3, both nominated from prior cistrome and/or tumor transcriptome studies [13, 14]. In our dataset, ROR1 displayed increased expression following NKX2-1 knockdown in one cell line (HCC1833), and was associated with a ChIP peak in two cell lines (H661 and H1195). LMO3 showed decreased expression in one cell line (HCC1195), and was associated with a ChIP peak in two cell lines (H1819 and HCC1195), and thus also did not meet our prioritization criteria.

From our integrative -omic analysis, EGFR emerged as a top candidate with obvious biological and clinical interest. *EGFR* is amplified and/or mutated in at least 10–15% of NSCLC (predominantly adenocarcimomas) [3], and those activating alterations predict clinical response to EGFR inhibitors including erlotinib and gefitinib [40]. *EGFR* mutations tend to occur in lung adenocarcinomas from female non-smokers [41]. In contrast, *KRAS* mutations, despite ostensibly functioning just downstream of EGFR, tend to occur in adenocarcinomas with mucinous histology from male smokers [3, 41]. Our findings suggest that *NKX2-1* amplification mediates its oncogenic effects at least in part through upregulation/activation of EGFR and its downstream signaling pathways (MAPK and PI3K).

Notably, our findings are consonant with prior observations from genetically-engineered mouse models. In particular, Nkx2-1 was recently shown to be required for Egfr-driven murine lung cancer [16]. As suggested from our data, Nkx2-1 might be required for the upregulated expression of Egfr transcript and/or protein (albeit the *Egfr* transgene is not present at its native genome locus). In contrast, in Kras mutant mice Nkx2-1 *loss* was shown to accelerate lung cancer (mucinous adenocarcinoma) [16] and potentiate lung cancer metastasis [15]. NKX2-1 likely plays distinct roles in the context of EGFR-driven and KRAS-driven lung cancers, with Egfr-driven murine tumors perhaps more closely modeling *NKX2-1* amplified human lung cancer.

**Fig 6:**
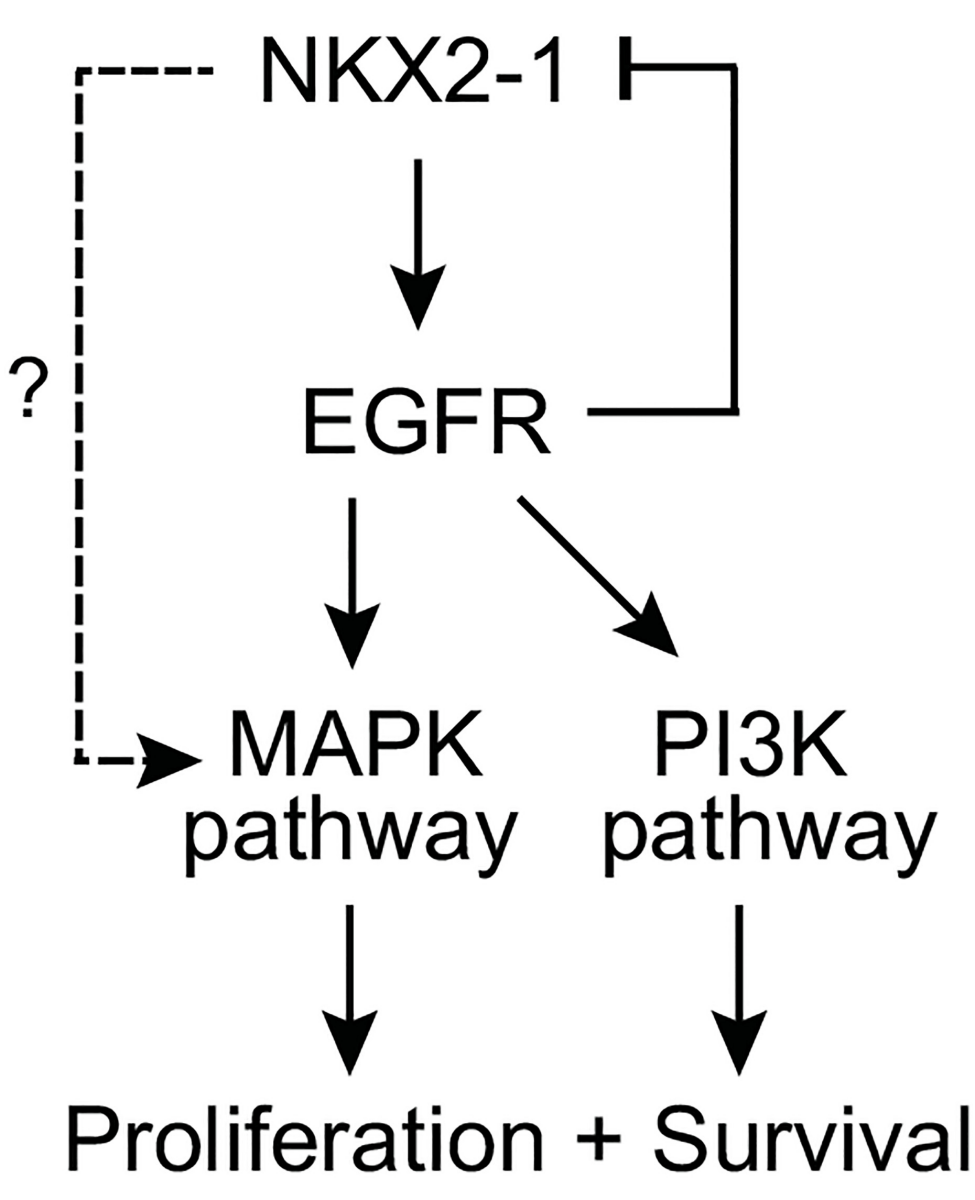
Model of EGFR as downstream mediator NKX2-1 oncogenic signaling. Schematic figure summarizes pathways and relationships deduced from experimental findings; see discussion in main text.

Our findings further suggest a feedback inhibition loop between NKX2-1 and EGFR, which regulates downstream signaling (summarized in Fig 6). In support of this, combined knockdown of NKX2-1 and EGFR further reduced MAPK and PI3K signaling, with resultant diminished cell proliferation. However, it is possible that NKX2-1 also transcriptionally regulates other inputs into MAPK and PI3K signaling (as suggested by our NKX2-1 binding site analysis; Table L in S1 File), and/or other pathways affecting cell proliferation and survival. Indeed, it is rather over-simplistic to presume that a transcription factor such as NKX2-1 acts through only a single downstream target (whether EGFR, or ROR1 or LMO3 as proposed by others). Moreover, NKX2-1 is subject to post-translational modification (e.g. phosphorylation and acetylation) affecting its activity [4] that could potentially influence its modulation of target genes and hence oncogenesis.

Notably, NKX2-1 knockdown enhanced the growth-suppressive effect of EGFR inhibition, whether by siRNA or by the small molecule inhibitor erlotinib. EGFR inhibitors including erlotinib are used clinically to treat lung cancers carrying activating EGFR mutations, but are less effective against lung cancers with wildtype EGFR [40]. Our findings suggest that concomitant NKX2-1 inhibition may augment the efficacy of EGFR inhibitors in *NKX2-1* amplified lung tumors. Although transcription factors are not considered ideal drug targets, recent successes in this area [42] suggest that future investigations based on this strategy may be warranted.

Further studies should define the precise mechanisms by which EGFR effectuates NKX2-1 oncogenesis in NSCLC. Nevertheless, our studies newly link two key NSCLC oncogenes, *NKX2-1* and *EGFR*, thereby uncovering an important new area of investigation and possible therapeutic strategy. Furthermore, the RNAseq and ChIPseq datasets should provide a rich resource for mining additional mediators and cooperators of NKX2-1 oncogenesis.

## Supporting Information

S1 File Supporting Information Tables. **Table A:** Dharmacon siRNAs. **Table B:** qPCR primers. **Table C:** RNAseq and ChIPseq read information. **Table D:** Genes measurably expressed across all 4 cell lines. **Table E:** Genes substantially altered following NKX2-1 knockdown. **Table F:** Genes with consistently altered expression following NKK2-1 knockdown. **Table G:** Canonical pathway gene sets found enriched by two-class GSEA. **Table H:** Canonical pathway gene sets enriched in each individual cell line. **Table I:** Top 100 up- and down-regulated genes in each cell line. **Table J:** Genes substantially downregulated with NKX2-1 knockdown in 3 cell lines. **Table K:** NKX2-1 binding site associated genes. **Table L:** Canonical pathway gene sets enriched among NKX2-1 binding site genes. **Table M:** Overlap of substantially regulated genes and binding-peak genes (Summary). **Table N:** Overlap of substantially regulated genes and binding-peak genes.
(XLSX)

S2 File Supporting Information Figures. **Figure A:** NKX2-1 isoform expression in *NKX2-1*-amplified NSCLC cell lines. (A) Schematic representation of the two NKX2-1 transcript variants and corresponding protein isoforms. (B) HCC1195 and HCC1833 cells predominantly express the short isoform of NKX2-1 protein, as determined by western blot co-migration with NKX2-1 short (and not long) isoform, exogenously expressed in 293T cells. **Figure B:** Independent siRNAs recapitulate findings from siRNA pools. (A, B) Compared to non-targeting control (NTC) siRNA, two independent siRNAs targeting NKX2-1 in H1819 cells result in (A) reduced NKX2-1 transcript levels by Q-RT-PCR (transcript levels normalized to GAPDH; error bars indicate max/min values), (B) reduced NKX2-1 protein levels by western blot (equal loading confirmed by Ponceau S staining; not shown), and (C) reduced cell proliferation by Wst-1 assay (*, *P*<0.05; ***P<0/001). (D, E) The two independent siRNAs targeting NKX2-1 also result in (D) reduced EGFR transcript levels, and (E) reduced EGFR protein levels. (F, G) Two independent siRNAs targeting EGFR result in (F) reduced EGFR transcript levels, and (G) reduced EGFR protein levels. (H) The two independent siRNAs targeting EGFR result in increased NKX2-1 protein levels. (I) EGFR knockdown does not alter NKX2-1 transcript levels, suggesting that the resultant increased NKX2-1 protein levels are the consequence of post-transcriptional regulation. **Figure C:** Supporting transcriptome data. (A) Gene numbers well-measured by RNAseq across all 4 NSCLC cell lines. (B) Gene numbers substantially (≥25%) downregulated (blue) or upregulated (red) in each of the 4 NSCLC cell lines. (C) Validation by Q-RT-PCR of select genes identified by RNAseq to be substantially downregulated following NKX2-1 knockdown. Q-RT-PCR transcript levels normalized to GAPDH; error bars indicate max/min values. **Figure D:** Supporting cistrome data. (A) Histogram showing distribution of NKX2-1 binding sites with respect to annotated genes (transcription start sites). (B) NKX2-1 binding peaks identified at the EGFR locus in H661, H1819, and HCC1195 cells. Called binding peaks are identified by blue triangles, and numbered. *Below*, the 100bp sequence of each NKX2-1 ChIPseq binding site is shown, highlighting NKX2-1 binding site motifs (CHCTY; see Fig 3C). **Figure E:** Integration of transcriptome and cistrome data. Shown for each of the 4 NSCLC cell lines is the overlap of NKX2-1 significantly-regulated genes and NKX2-1 binding site-associated genes. **Figure F:** EGFR protein levels and erlotinib response in NSCLC lines. (A) EGFR protein levels in *NKX2-1* amplified/dependent NSCLC cell lines. EGFR protein levels quantified by western blot; α-tubulin serves as a loading control. (B) Erlotinib dose-response curve in H1819 cells. Fifty percent growth inhibitory concentration (IC_50_) is indicated.
(PDF)
