# Supplementary figures and images for: Integrative Genomics Implicates EGFR as a Downstream Mediator in NKX2-1 Amplified Non-Small Cell Lung Cancer
Source: PLoS One. 2015 Nov 10;10(11):e0142061. doi: 10.1371/journal.pone.0142061 (PMC4640868; doi:10.1371/journal.pone.0142061)

Figure A

A

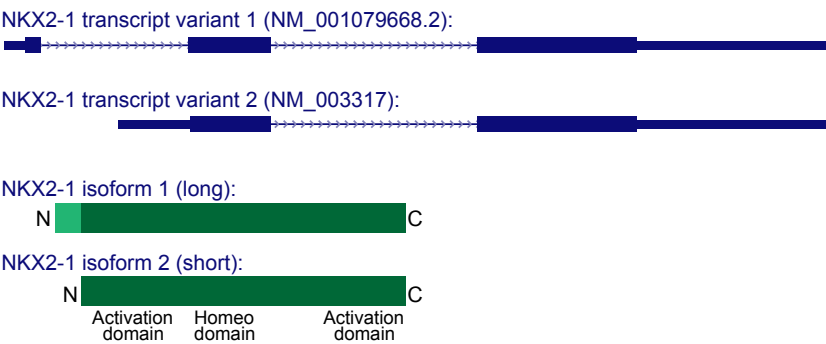

B

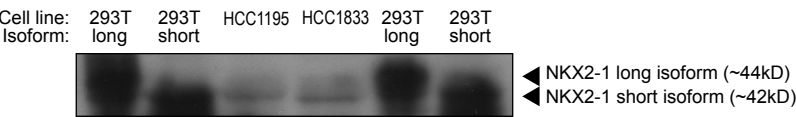

Figure B

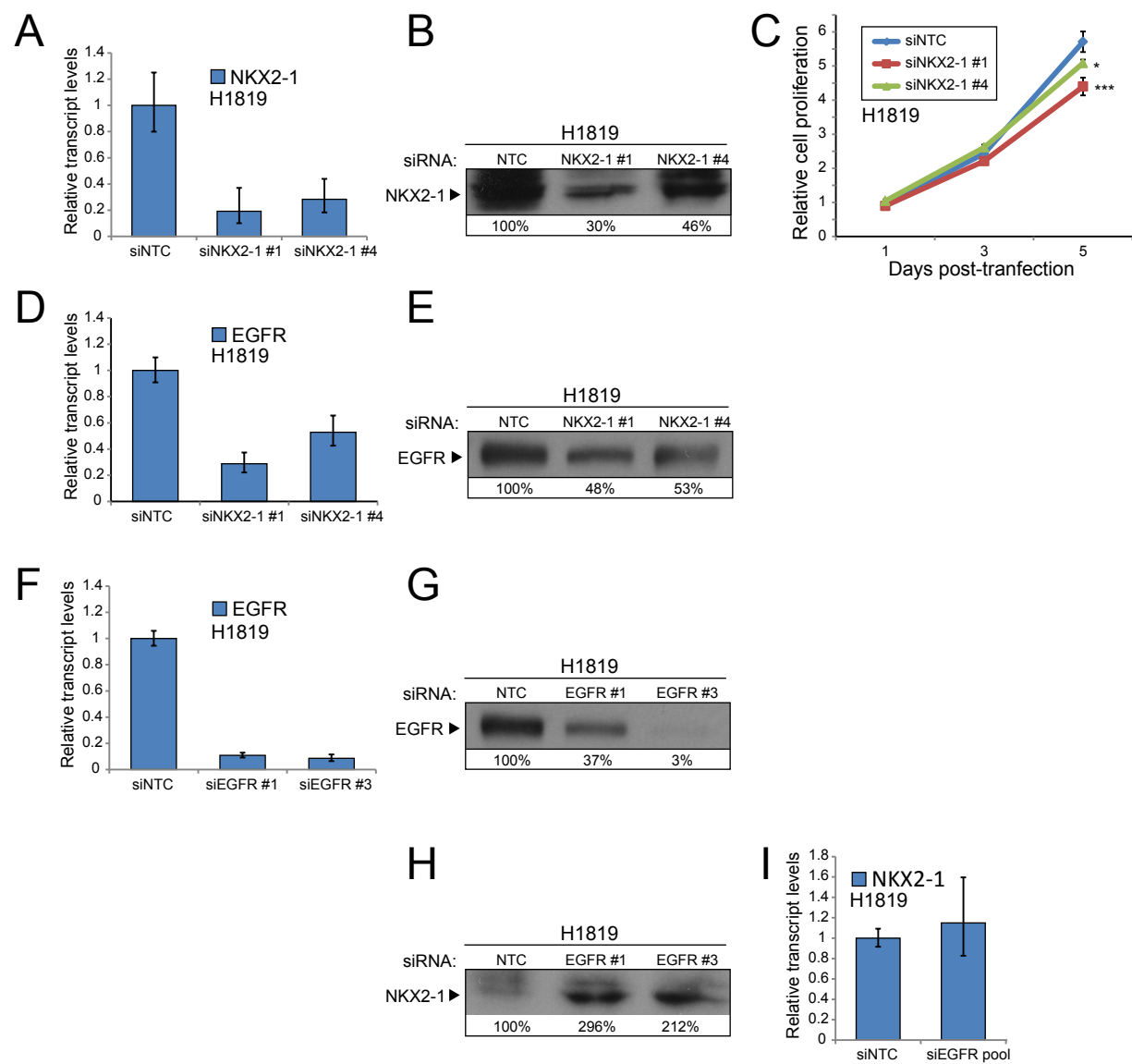

Figure C

A

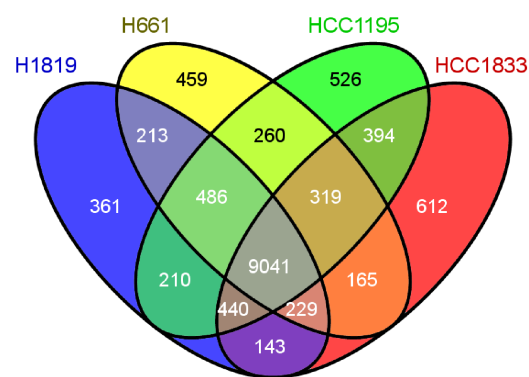

B

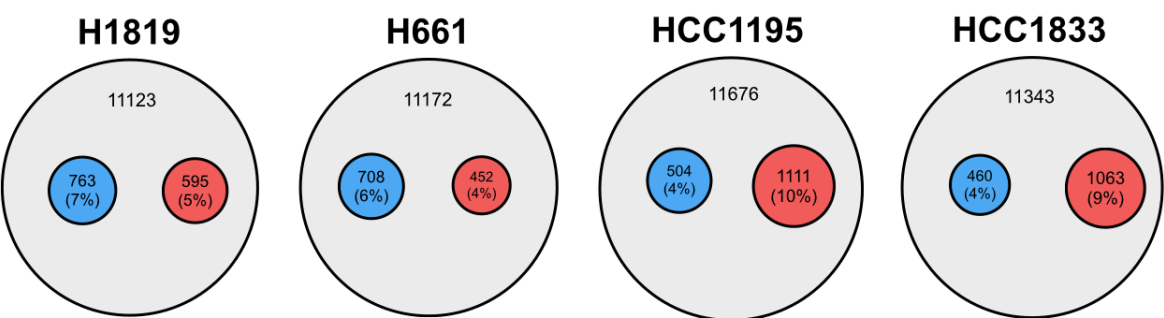

C

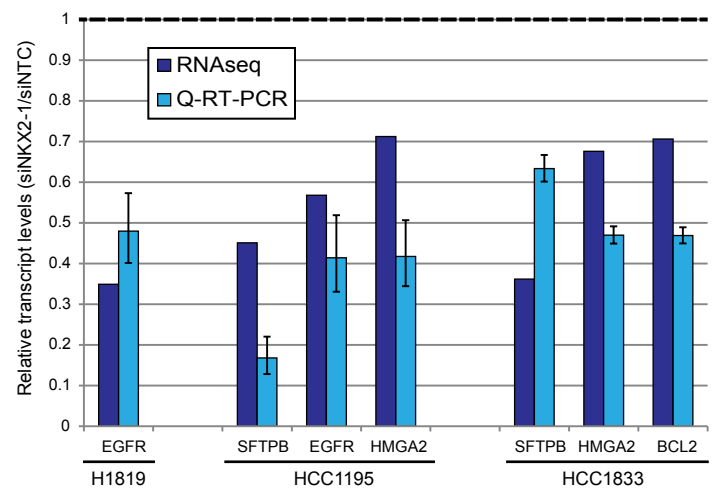

Figure D

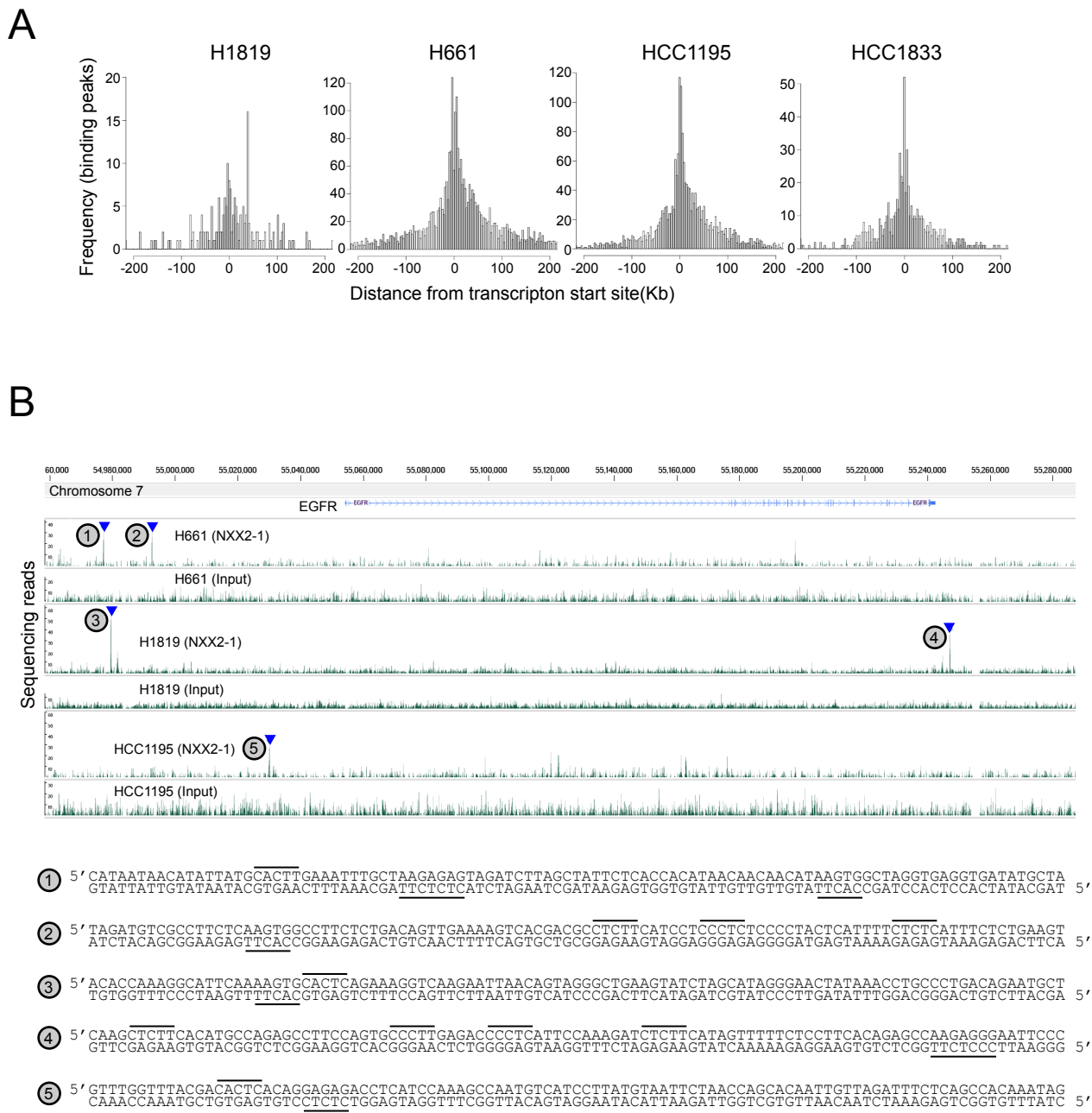

Figure E

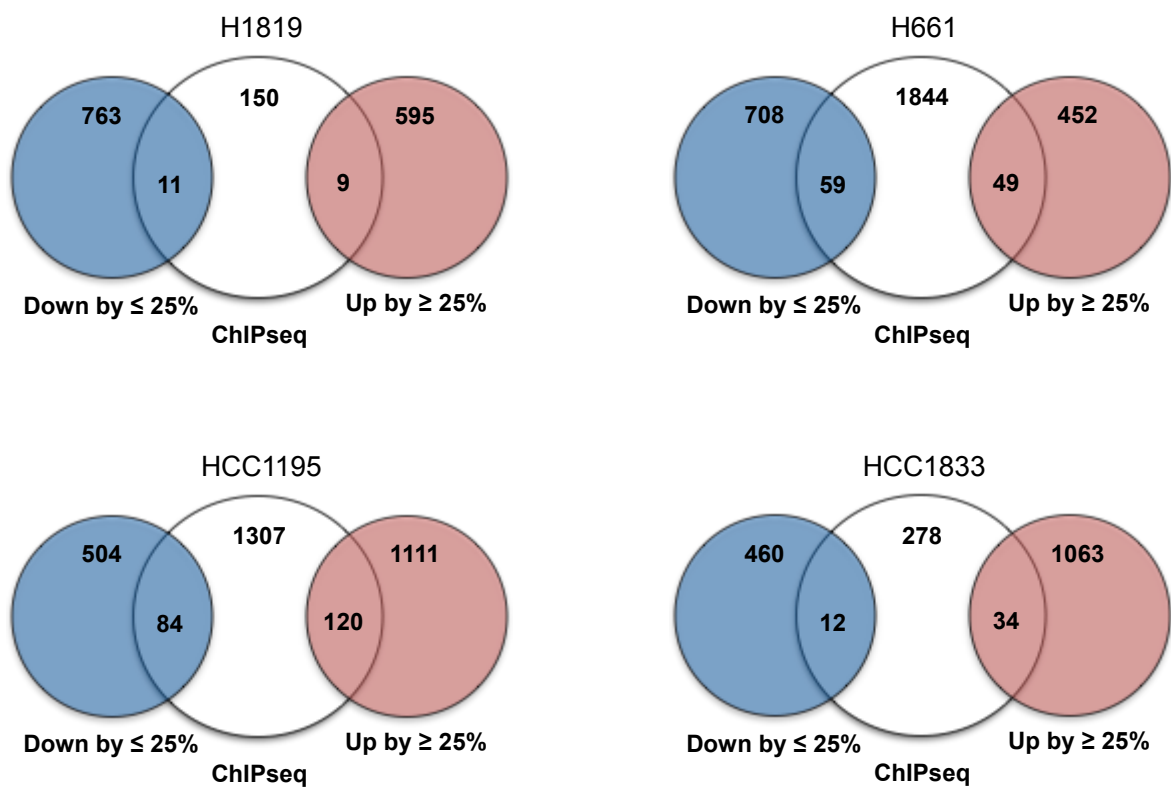

Figure F

A

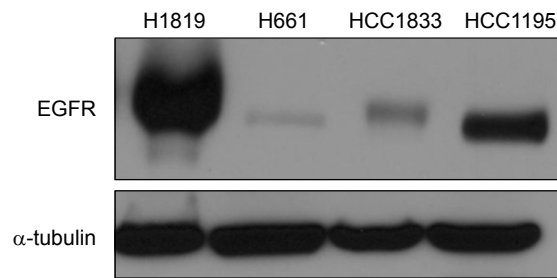

B

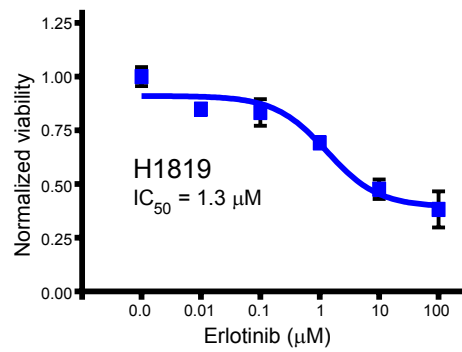

Supplement: S2 File — Figure A: NKX2-1 isoform expression in NKX2-1-amplified NSCLC cell lines. (A) Schematic representation of the two NKX2-1 transcript variants and corresponding protein isoforms. (B) HCC1195 and HCC1833 cells predominantly express the short isoform of NKX2-1 protein, as determined by western blot co-migration with NKX2-1 short (and not long) isoform, exogenously expressed in 293T cells. Figure B: Independent siRNAs recapitulate findings from siRNA pools. (A, B) Compared to non-targeting control (NTC) siRNA, two independent siRNAs targeting NKX2-1 in H1819 cells result in (A) reduced NKX2-1 transcript levels by Q-RT-PCR (transcript levels normalized to GAPDH; error bars indicate max/min values), (B) reduced NKX2-1 protein levels by western blot (equal loading confirmed by Ponceau S staining; not shown), and (C) reduced cell proliferation by Wst-1 assay (*, P<0.05; ***P<0/001). (D, E) The two independent siRNAs targeting NKX2-1 also result in (D) reduced EGFR transcript levels, and (E) reduced EGFR protein levels. (F, G) Two independent siRNAs targeting EGFR result in (F) reduced EGFR transcript levels, and (G) reduced EGFR protein levels. (H) The two independent siRNAs targeting EGFR result in increased NKX2-1 protein levels. (I) EGFR knockdown does not alter NKX2-1 transcript levels, suggesting that the resultant increased NKX2-1 protein levels are the consequence of post-transcriptional regulation. Figure C: Supporting transcriptome data. (A) Gene numbers well-measured by RNAseq across all 4 NSCLC cell lines. (B) Gene numbers substantially (≥25%) downregulated (blue) or upregulated (red) in each of the 4 NSCLC cell lines. (C) Validation by Q-RT-PCR of select genes identified by RNAseq to be substantially downregulated following NKX2-1 knockdown. Q-RT-PCR transcript levels normalized to GAPDH; error bars indicate max/min values. Figure D: Supporting cistrome data. (A) Histogram showing distribution of NKX2-1 binding sites with respect to annotated genes (transcription [file pone.0142061.s002.pdf]
